# Supplementary material for: Out-of-plane orientation of luminescent excitons in two-dimensional indium selenide
Source: Nat Commun. 2019 Sep 2;10:3913. doi: 10.1038/s41467-019-11920-4 (PMC6718420; doi:10.1038/s41467-019-11920-4)
Supplement: Supplementary file 1 — Supplementary Information [file 41467_2019_11920_MOESM1_ESM.pdf]

Supplementary Information for

Out-of-plane orientation of luminescent excitons in two-dimensional indium selenide

Mauro Brotons-Gisbert,* Raphaël Proux, Raphaël Picard, Daniel Andres-Penares, Artur Branny, Alejandro Molina-Sánchez, Juan F. Sánchez-Royo* and Brian D. Gerardot*

*Correspondence to:

M.Brotons_i_Gisbert@hw.ac.uk; Juan.F.Sanchez@uv.es; B.D.Gerardot@hw.ac.uk

Supplementary Figure 1

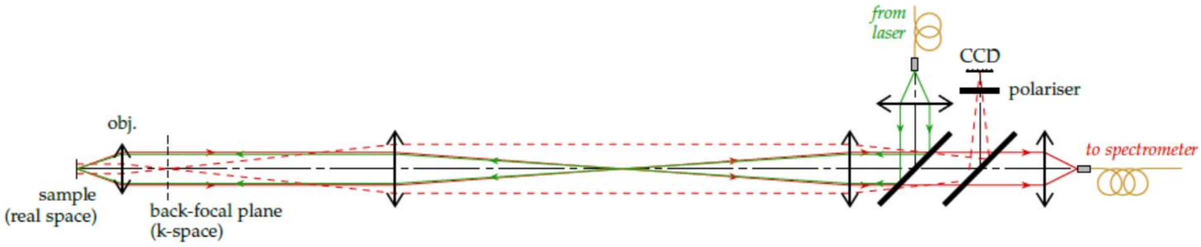

Supplementary Figure 1. Sketch of the microscope setup used for both photoluminescence measurements and back-focal plane imaging. A flip mirror is used to shift from the photoluminescence to the back-focal plane imaging configuration.

Supplementary Note 1: Orientation of luminescent dipoles in ML $MoSe_2$

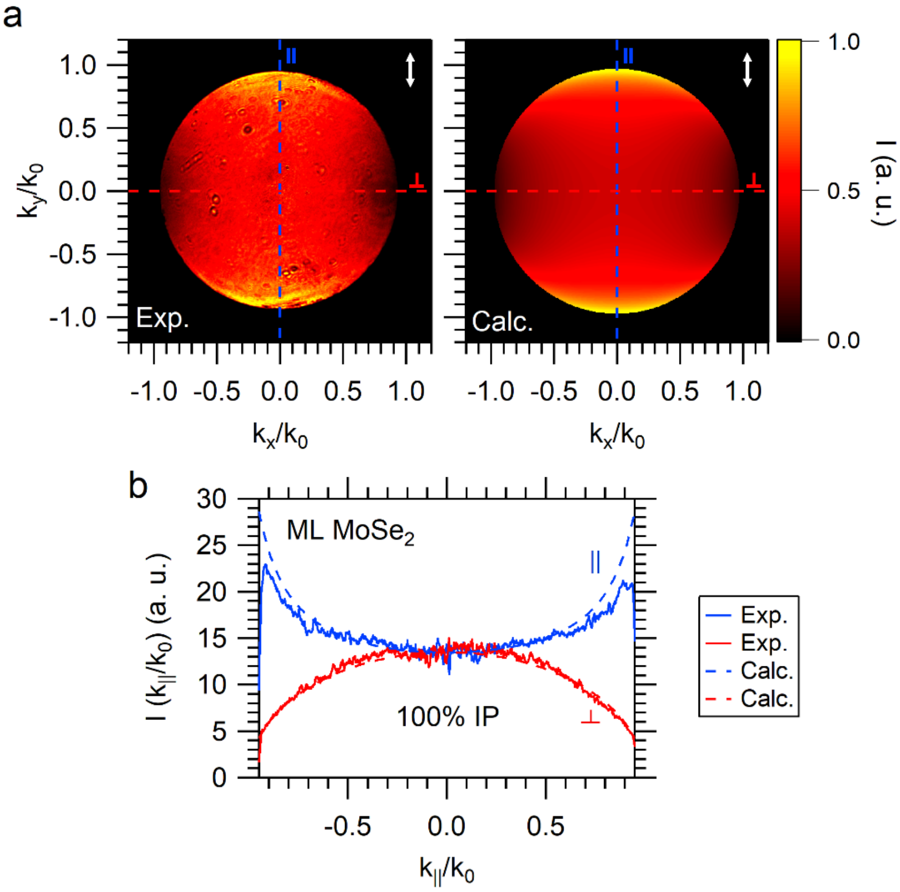

Supplementary Figure 2. Orientation of luminescent dipoles in ML $MoSe_2$. a, Experimental (left panel) and calculated (right panel) **k**-space images of the photoluminescence signal of ML $MoSe_2$ deposited on top of a gold substrate. **b**, Experimental (solid lines) and calculated (dashed lines) **k**-spaces profiles measured for directions perpendicular (red) and parallel (blue) to the detection polariser.

Supplementary Figure 2a shows the results of the **k**-space imaging study carried out on a ML $MoSe_2$ flake deposited on top of a gold substrate. The left panel shows the normalised experimental **k**-space emission pattern, while the right panel shows the corresponding calculated **k**-space pattern assuming a 100% IP orientation of luminescent dipoles in $MoSe_2$. The vertical (k_y/k_0) and horizontal (k_x/k_0) axes represent the orthogonal components of the in-plane photon wavevector ($k_{||} = k_0\sin(\theta)$, with θ being the emission angle) normalised to the photon wavevector in air (k_0). Radiation with a wavevector $|k_{||}|$ larger than the numerical aperture of the objective is not collected by the objective (black region). The white arrow in the top right corner indicates the orientation of the transmission axis of the linear polariser used during the experiment. The vertical and horizontal cross-sections of the experimental and calculated **k**-space emission patterns are shown in Supplementary Figure 2b. A very good agreement can be observed between the experimental and calculated **k**-space profiles, confirming a 100% IP intrinsic dipole distribution for ML $MoSe_2$.

Supplementary Note 2: Photoluminescence of ML WSe_2

The ML thickness of the WSe_2 flake used in our experiments was confirmed by means of room-temperature photoluminescence measurements. Supplementary Figure 3 shows the room temperature PL of a ML WSe_2 deposited on top of a SiO_2/Si substrate with a SiO_2 thicknesses of 104 nm. The shape and energy positions of the PL peak confirm the ML thickness of the flake.

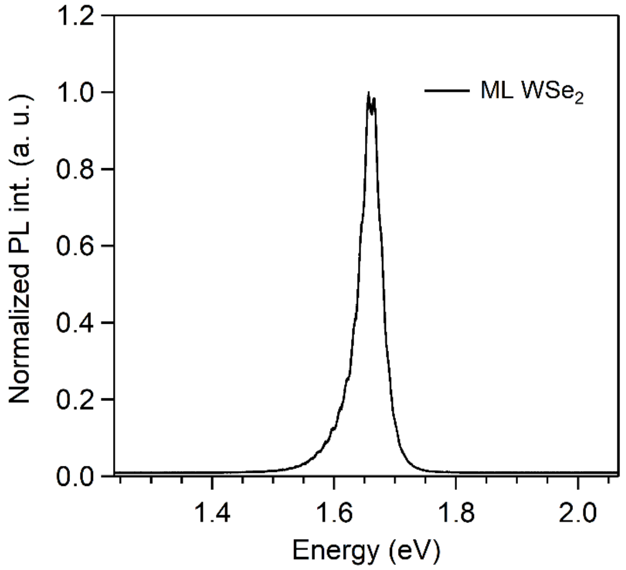

Supplementary Figure 3. Room-temperature photoluminescence of ML WSe_2.

Supplementary Note 3: Bethe-Salpeter Equation and convergence of the exciton binding energy of bulk InSe

The optical spectra including excitonic effects are obtained with the formalism of the Bethe-Salpeter Equation (BSE) (references in the main text). The accurate calculation of the excitonic binding energy in bulk InSe requires a full study of convergence that is out of the scope of our work. Nevertheless, we have performed a convergence study to demonstrate that the OP/IP absorption ratio has already converged with the **k**-grid employed in our calculations (15x15x10), which implies that the conclusions of our work are well supported by the calculations. Supplementary Figure 4 shows the OP (solid lines) and IP (dashed lines) absorption spectra for an increasing **k**-point sampling in logarithmic scale. We find that the OP/IP absorption ratio is almost constant for an increasing number of **k**-points beyond (12x12x4).

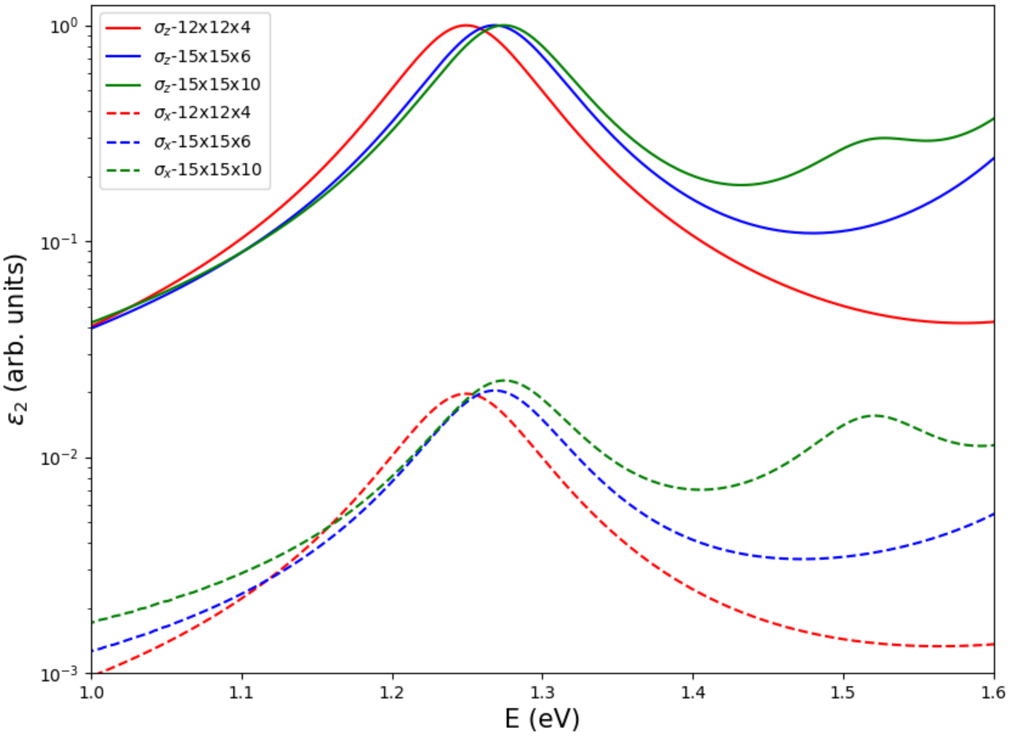

Supplementary Figure 4. Calculated IP and OP optical absorption for several k-grids.

In addition, we have checked the convergence of the exciton binding energy. Supplementary Figure 5a shows the calculated optical absorption of bulk InSe for an increasing number of k-points (the critical parameter to calculate the exciton binding energy). The binding energy as a function of the number of **k**-points (with the specific **k**-grid annotated next to the points) is shown in Supplementary Figure 5b. From the data we can conclude that a full convergence would lead to a value close to the experimental value of 14 meV.

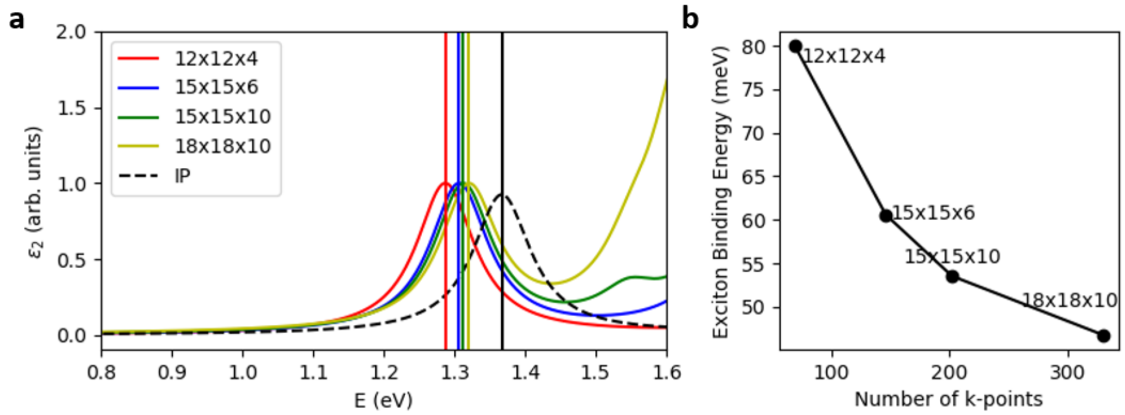

Supplementary Figure 5: Dependence of the calculated binding energy of bulk InSe as a function of the K-grid employed. (**a**) Calculated optical absorption of bulk InSe for an increasing number of **k**-points. (**b**) Calculated binding energy as a function of the number of **k**-points.
